# Programmed cell death and redox metabolism protect *Chlamydomonas reinhardtii* populations from the galactic cosmic environment on the Artemis-1 mission

**DOI:** 10.1038/s41598-025-05419-w

**Published:** 2025-07-02

**Authors:** Timothy G. Hammond, Sajanlal R. Panikkanvalappil, Patricia L. Allen, Hamid Kian Gaikani, Corey Nislow, Guri Giaever, Ye Zhang, Howard G. Levine, Ramona Gaza, Dinah Dimapilis, Howard W. Wells, James M. Russick, Pierre M. Durand, Holly H. Birdsall

**Affiliations:** 1 Research Service Line, Durham VA Health Care System, Building 15, Room 210 508 Fulton Street, Durham, NC 27705 USA; 2 Nephrology Division, Department of Internal Medicine, Duke University School of Medicine, Durham, NC 27705 USA; 3 Department of Imaging, Dana-Farber Cancer Institute, Harvard Medical School, Boston, MA USA; 4 Department of Radiology, Brigham & Women’s Hospital, Harvard Medical School, Boston, MA USA; 5 Institute for Medical Research, Durham, NC 27705 USA; 6 Faculty of Pharmaceutical Sciences, The University of British Columbia, Vancouver, BC V6T 1Z3 Canada; 7 Kennedy Space Center, NASA-Kennedy Space Center, Utilization & Life Sciences Office, Merritt Island, FL 32899 USA; 8 Civil Group Integrated Missions Operation, Leidos, Houston, TX 77258 USA; 9 Space Radiation Analysis Group, NASA Johnson Space Center, Houston, TX 77058 USA; 10 Kennedy Space Center, The Bionetics Corporation, Mail code: Bio-2, Florida, FL 32899 USA; 11 Evolutionary Studies Institute, University of the Witwatersrand, Johannesburg, 2193 South Africa; 12 Departments of Otorhinolaryngology, Immunology, and Psychiatry, Baylor College of Medicine, Houston, TX 77030 USA; 13 Research Service Line, Durham VA Health Care System, Durham, NC 27705 USA

**Keywords:** Cosmic radiation, *Chlamydomonas reinhardtii*, Carotenoids, Tardigrade, Spaceflight, Artemis-1, Cell biology, Microbiology

## Abstract

On the Artemis I mission, *Chlamydomonas reinhardtii*, a green unicellular flagellate alga, was exposed to the galactic cosmic environment. A new flight hardware termed “Moonshot” was designed, built, and flown. “Moonshot” performed flawlessly, and is available as flight-certified, flight-proven hardware for timed illumination and monitoring for flight and terrestrial applications. The *Chlamydomonas* strains were spotted on nutrient agar plates and flown on Artemis I in the new Moonshot hardware that provided six hours of light daily to synchronize the algal cell cycle and tracked temperature, power use, and gravity over time. Synchronous ground controls in identical hardware were run in parallel. The Artemis-1 flight of *Chlamydomonas reinhardtii* around the Moon with exposure to the galactic cosmic environment showed: (1) Flown samples exposed to cosmic radiation showed increased programmed cell death and decreased necrosis compared to ground control samples. (2) There was robust *Chlamydomonas* growth in both flown and ground control samples post flight. (3) Raman spectroscopy analysis showed that redox-protective terpenoid carotene pigments, known cell death mediators, were increased during flight around the moon. (4) Insertion of the *Dsup* tardigrade gene was protective both on the ground and in flight.

## Introduction

The study of the biological effects of the galactic cosmic environment has a dual purpose. First, understanding the biology of galactic cosmic environment should guide development of protective measures for astronauts flying beyond low Earth orbit^1^, as well as leveraging biological systems to produce meaningful biologics in the space environment^2,3^.

On planned missions to the Moon and Mars, cosmic radiation exposure is considered a severe risk to astronauts, plant crops, and biologics due to the potential DNA damage inflicted by the high energy nature of cosmic particles^4^. Modalities protecting from cosmic radiation damage are urgently needed. To this end, several lines of evidence show that multiple tardigrade genes, including the intrinsically disordered protein Dsup, afford protection from a diverse array of stresses including various forms of radiation present in low Earth orbit^5,6^.

Experiments flown on the Artemis 1 mission that circumnavigated the Moon, represent the first opportunity since the Apollo era to return to Earth biological samples exposed to the galactic cosmic environment (https://www.nasa.gov/reference/artemis-i/). To understand the novelty of this opportunity, it is critical to remember that the International Space Station (ISS) is inside the radioprotective Van Allen Belts. Although experiments and personnel on the ISS are exposed to microgravity, reduced convection, and more radiation than on Earth^7^, they are shielded from the vast majority of galactic cosmic radiation by the Van Allen Belts^8^.

The green alga *Chlamydomonas reinhardtii* was selected as the biological model system. It is a well-characterized, motile, single-celled green alga whose genome is fully sequenced and is relatively easy to engineer molecularly. During previous growth in space, a light-dependent increase in photosystem II has been observed^9^. Programmed cell death (PCD) is sometimes a population survival mechanism in microalgae^10–12^.

The primary hypothesis was that exposure to galactic cosmic environment would reduce *Chlamydomonas* survival through unregulated cell death mechanisms called necrosis. An alternative balancing argument was that the radiation stimulus would induce a population survival response via PCD. There are various conceptualizations of PCD especially as it pertains to unicellular organisms. In our study we use a general mechanistic understanding of PCD as an active, genetically based mechanism of regulated cell destruction^13^ that is distinguishable from incidental forms of death such as cell necrosis. In unicellular organisms, including *Chlamydomonas*, the externalization of phosphatidylserine on the cellular membrane, detectable by Annexin binding, has been utilized to assay PCD^10^.

Measurements on the galactic cosmic environment exposed *Chlamydomonas*, and ground-based controls, included mechanisms of cell death, and protection of tardigrade *Dsup* transformation. An aliquot of the green algae strains flown in this experiment had the tardigrade gene *Dsup* inserted into the cell nucleus to test for protection from galactic cosmic environment.

Raman spectroscopy was used for real-time chemical analysis of the *Chlamydomonas* cells^14,15^. The Curiosity rover on Mars uses fluorescence and Raman spectroscopy to search for organic molecules as a possible sign of life^16^, and ESA has analyzed biological samples returned from the ISS using this technology^17,18^. But the potential of the biological application of these techniques is only starting to be realized^14,15^. Raman spectroscopy is a non-destructive and non-invasive technique that offers several advantages in studying various stress-induced (including radiation) biomolecular modifications at the single-cell level without altering their integrity^15,19^. Moreover, the sensitivity of Raman spectroscopy enables the detection of subtle changes in cellular redox metabolism, which is fundamental in elucidating the dynamic alterations in the levels of redox-active species, as well as the cellular response to oxidative stress caused by cosmic radiation.

The initiative began with the design and fabrication of affordable new flight hardware (named Moonshot) to support the growth of *Chlamydomonas reinhardtii*^20^, while simultaneously meeting the constraints of the Artemis I mission profile (https://www.nasa.gov/mission/artemis-i/).

## Results

**Fig. 1:**
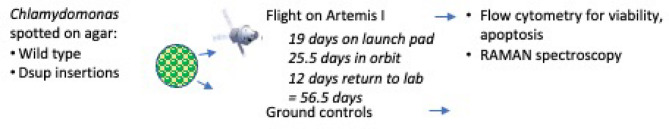
Schematic of experiments. A summary diagram of *Chlamydomonas* preparation, flight details, and analysis modalities.

The *Chlamydomonas* strains were spotted on nutrient agar plates and flown on Artemis I in the new Moonshot hardware that provided six hours of blue and red light daily to synchronize the algal cell cycle, and tracked temperature, power use, and gravity over time. Synchronous ground controls in identical hardware were run in parallel (Fig. 1) in a temperature-controlled incubator adjusted daily to mirror the Artemis I profile.

### Moonshot hardware

The Moonshot hardware performed nominally throughout the flight and ground control experiments. The maximum temperature delta between flight and ground control hardware copies was 1.4^o^C, well within the envelope of tolerable experimental conditions. At the end of the 25.5-day of flight, the batteries still had more than 50% of power remaining. The power profile validated that the lights came on for 6 out of 24 h daily, as planned. Acceleration recordings show serial measurements of gravity in the z axis, but no acceleration in other directions, as expected. The hardware met all the validation criteria for power use, power cycling, temperature control, g-force recording in three axes, data capture, and downloading for analysis, plus materials, and biological compatibility. The Moonshot hardware is now not only flight certified but flight proven.

### Radiation exposure in assorted environments in space and on earth

The total radiation exposure during the flight as measured in the RAM and CAD are reported in Table 1. Comparison of the exposure levels during flight to other environmental systems (also shown in Table) is examined in the discussion section.

**Table 1:** Radiation exposure in assorted environments in space and on earth.

Exposure	Radiation (mGy)	Ref.
Radiation Area Monitor Artemis I Mission Dose	11.4 ± 0.4	This study
Crew Active Dosimeter Artemis I Mission Dose	12.6 ± 0.5	This study
Naturally occurring “background” for 25.5 days (Earth)	0.17	^51^
CAT-Scan of Abdomen & Pelvis ± contrast	15.4	^52^
Exposure on Mars for 25.5 days (Curiosity rover)	5.4	^50,62^
Mars transit for 25.5 days (Curiosity rover)	11.8	^50,62^
International Space Station for 25.5 days	5.7	^7^

Comparison of total radiation doses for 25.5 days during the Artemis I mission, plus 25.5 days of exposure to background radiation on earth. the curiosity Rover in transit to Mars, and on Mars, and 25.5 days on the ISS. CAT-scan exposure is a one-time dose.

### Growth and microscopic appearance postflight

**Fig. 2:**
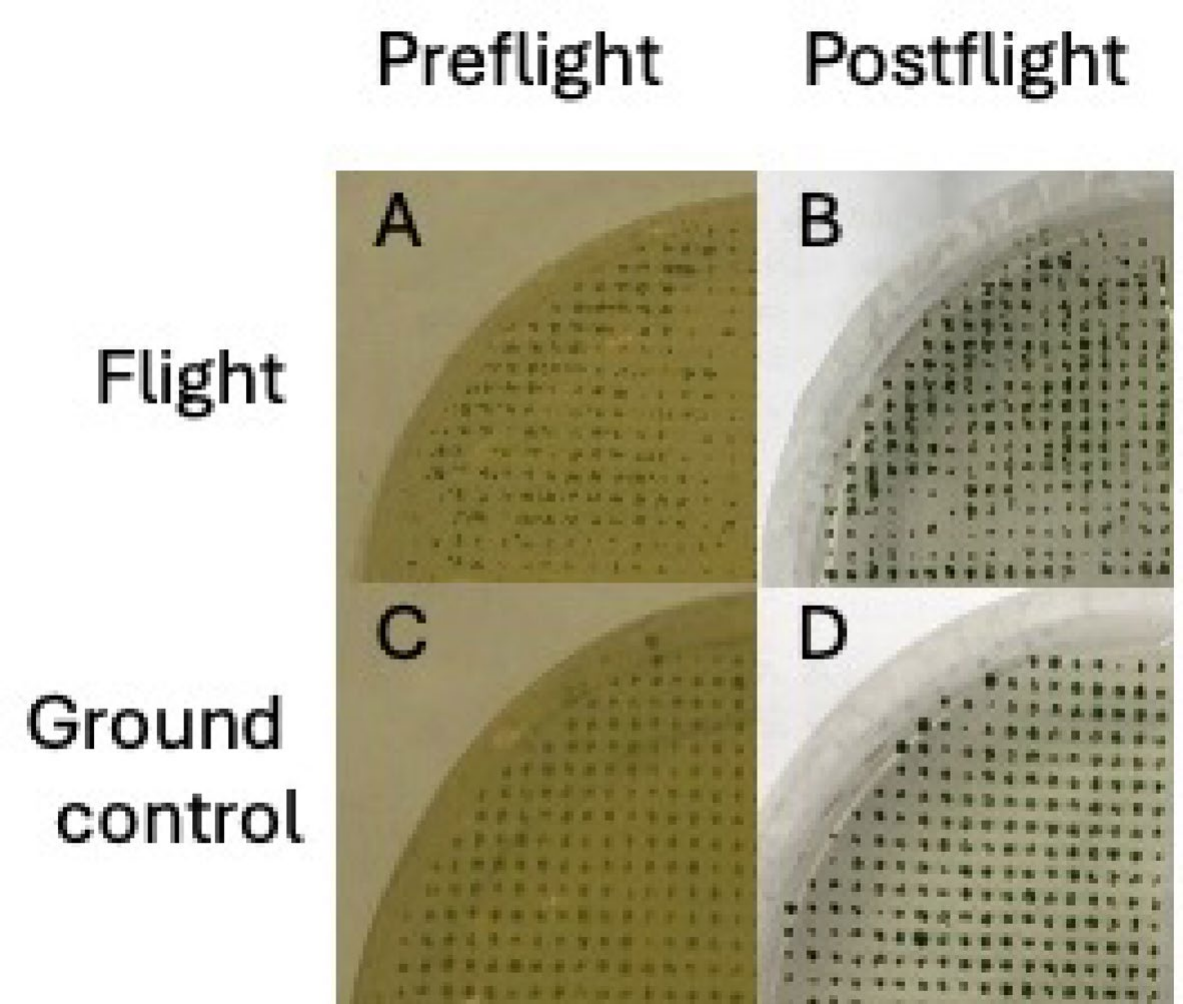
Comparison of *Chlamydomonas* colonies before and after flight. Photograph of a spotted plate before flight (Fig. 2A on the upper left) and after flight (Fig. 2B on the upper right), as well as photograph of a spotted ground control plate before flight (Fig. 2c on the lower left) and ground control plate after flight (Fig. 2d on the lower right),

Post flight, colonies were slightly larger and more intensely green (Fig. 2), with flight and ground samples visually indistinguishable. Three aliquots each of ground and flight samples inoculated into fresh liquid TAP grew robustly over time. While initially rare, swimming algae were observed in the cultures by light microscopy, and the percentage of swimmers increased as the cultures grew.

### Flow cytometry assay of viability and PCD of *Chlamydomonas* in flight vs. ground

**Fig. 3:**
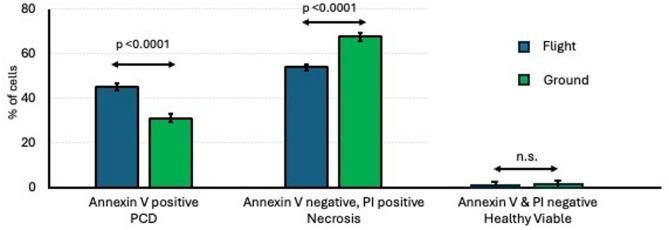
Viability and programmed cell death (PCD) of *Chlamydomonas* in flight vs. ground. Aliquots of flown and ground control *Chlamydomonas* were stained with Annexin V, to identify PCD, and propidium iodide, to identify dead cells, and analyzed by flow cytometry. Values shown are the % positively staining cells and are the mean ± SEM of six replicates. The p values for significance were evaluated by unpaired two-tailed t-test.

The percentages of PCD cells (Annexin V positive), dead cells (propidium iodide positive), and healthy viable cells (Annexin V negative and propidium iodide negative) in the flight and ground control samples are shown in (Fig. 3). 45.1 ± 1.4% of the cells in flight had undergone PCD compared to 31.1 ± 2.0% of the cells in the ground control (mean ± standard error, *n* = 6, *p* < 0.001). 53.9 ± 1.3% of the cells in flight were necrotic (PI positive but not Annexin-V positive) compared to 67.6 ± 1.9% of the cells in the ground control (mean ± standard error, *n* = 6, *p* < 0.001). ~1% of the cells were healthy viable cells (PI negative and Annexin-V negative) in both groups (1.3 ± 0.2 ground versus 1.0 ± 0.2 in flight; no different).

### Flow cytometry assay of the effect of *Dsup* on the viability of *Chlamydomonas* in flight vs. ground

**Fig. 4:**
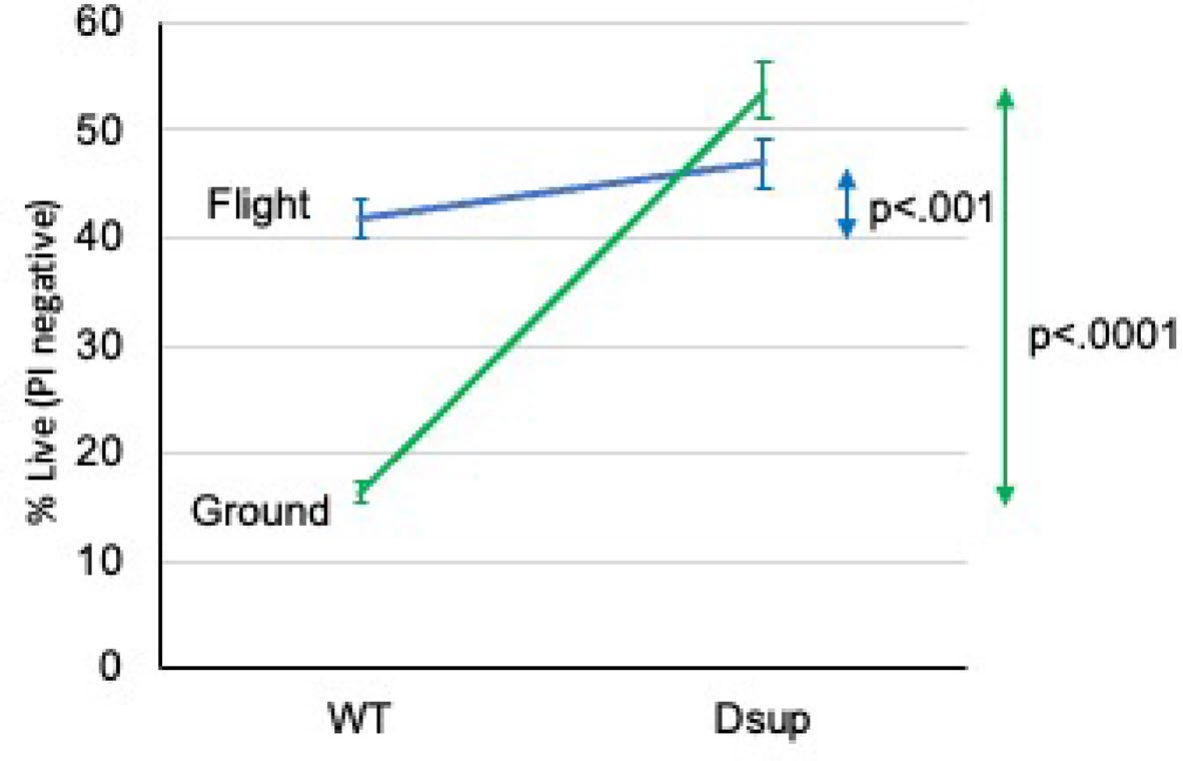
Effect of Dsup on the viability of *Chlamydomonas* in flight vs. ground. *Chlamydomonas* with insertion of the *Dsup* gene were compared to wild type (WT) strains in flown versus ground control samples. Aliquots of each were stained with propidium iodide and analyzed by flow cytometry. Values shown are the percent of viable cells, defined as excluding propidium iodide, and are the mean ± SEM of six replicates. The p values for significance were evaluated by unpaired two-tailed t-test.

The percentages of live cells (PI negative) in flown and ground control *Chlamydomonas* are shown in Fig. 4. 41.8 ± 1.7% of the flown cells were healthy viable or PCD compared with 46.9 ± 2.3% of the flown cells with *Dsup* insertion (mean ± standard error, *n* = 6, *p* < 0.001). 16.4 ± 0.9% of the ground control cells were healthy viable or PCD compared with 53.7 ± 2.6% of the ground control cells with *Dsup* insertion (mean ± standard error, *n* = 6, *p* < 0.001).

### Single-cell confocal Raman spectroscopy of *Chlamydomonas reinhardtii* in flight vs. ground

**Fig. 5:**
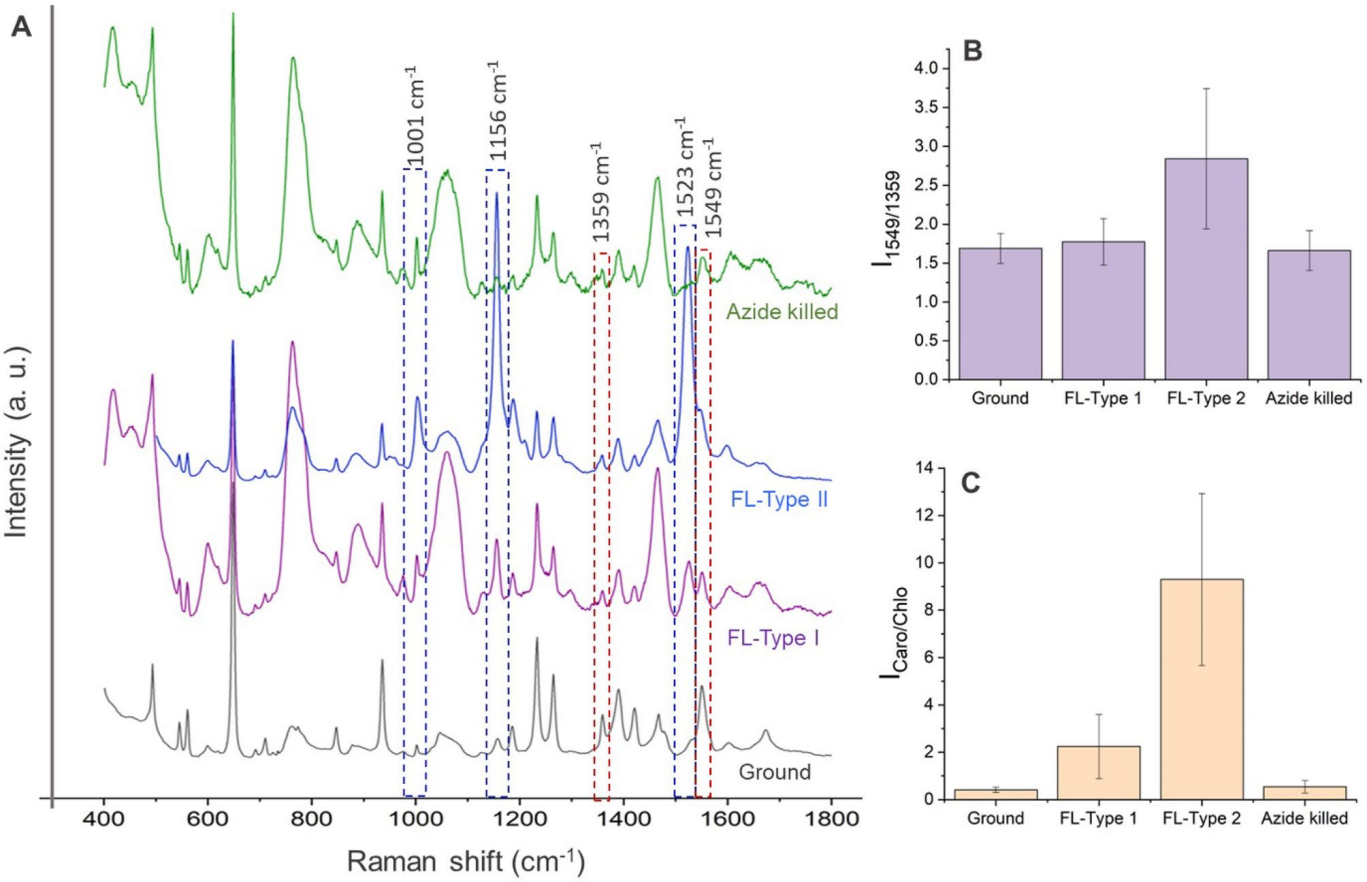
Raman spectra from ground control *Chlamydomonas* and Type I, Type II, and azide-killed cells from flown *Chlamydomonas*. Raman spectra of *Chlamydomonas* under different conditions are shown in **A**. Prominent Raman shifts at 1001, 1156, 1359, 1523, and 1549 cm^− 1^, associated with various molecular vibrations, are highlighted with dashed boxes. Histograms showing the ratio of the intensities of the Raman bands at 1549 cm^− 1^ and 1359 cm^− 1^ (I_1549/1359_) for various samples are shown in **B**. Flown *Chlamydomonas* contained two different cell types (Type I and Type II) with distinct Raman spectral features. The intensity of Raman vibration corresponding to carotenoids in Type I cells (FL-Type I) was much weaker. Whereas, in Type II cells (FL-Type II), characteristic carotenoid vibrations enhanced significantly. This ratio provides the relative chlorophyll content across different conditions. Histogram illustrating the carotenoid to chlorophyll ratio (I_Caro/Chlo_) across the various sample types are shown in **C**. The data points represent the mean value and error bars indicate the standard deviation, reflecting the variability of the response in each sample type. These spectra and ratios provide insights into the biochemical adaptations of *Chlamydomonas* to galactic radiation and microgravity, with significant variations noted between the two cell types we found in live flight samples, suggesting differential stress responses and survival strategies.

In this pioneering use of Raman spectroscopy for analyzing biological samples exposed to the galactic cosmic environment beyond low Earth orbit, we noticed a differential survival strategy among *Chlamydomonas* exposed to cosmic rays and microgravity. Specifically, we found two different cell types (Type I and Type II) with distinct Raman spectral features in flight (FL) samples compared to the ground samples. The intensity of Raman vibration corresponding to carotenoids in Type I cells (FL-Type I) was much weaker. Whereas, in Type II cells (FL-Type II), characteristic carotenoid vibrations were enhanced significantly (Fig. 5A). In Raman spectra of *Chlamydomonas*, the presence of prominent vibrations at 1001, 1156, and 1523 cm^− 1^ indicate the presence of carotenoids^21–24^, which are attributed to C–CH_3_ deformation, C–C stretching, and C =C stretching vibrations of polyene, respectively^23,25^. Considering the well-documented abundance of beta-carotene in *Chlamydomonas* and its established role in redox protection, along with the fact that the observed Raman bands correspond to the prominent vibrational modes characteristic of beta-carotene, we hypothesize that beta-carotene contributes significantly to the observed carotenoid Raman bands. The significant variation in these carotenoid Raman vibrations found in the spectral profiles between Type I and Type II algal cells offers a compelling insight into the heterogeneity of stress responses within a single-cell population in two-cell populations. The reduction in significant carotenoid vibrations in the Type I flight sample implies that these cells may not have been able to induce the same protective response and might have yielded to radiation-induced damage. Moreover, the azide-killed flight samples exhibited nearly identical Raman spectra with weak carotenoid signal as in Type I cells, corroborating our proposed link between the reduction in carotenoid and cell death pathways. The weaker carotenoid signals in these samples suggest that the living cells in the flight environment actively maintain or increase their carotenoid content. In contrast, the azide treatment or cell death ceases metabolic activity, thereby reducing the carotenoid levels.

It is possible and logical to propose that the two Raman populations (Type I and Type II) represent the flow cytometry populations of PCD cells and necrotic cells. However, we could not directly link or exclude that the flow cytometry PCD and necrotic populations are the Ramon spectroscopy Type I and Type II populations.

For better understanding the relative increase in the carotenoid content at various conditions, the intensity ratio (I_Caro/Chlo_) of carotenoid-chlorophyll vibration (1523 cm^− 1^ and 1359 cm^− 1^, respectively) was used (Fig. 5C). We also used Raman spectroscopy to identify any significant changes in chlorophyll composition by analyzing prominent and characteristic Raman bands of chlorophylls found at 1359 (vibrations of C–C and C–N bonds within the porphyrin ring) and 1549 cm^−^1 (primarily involves the C=C stretching vibrations within the porphyrin ring)^26^. We noticed that the intensity ratio (Fig. 5B) of these bands (I_1549/1359_) in flight samples (FL-Type I and FL-Type II) as well as in azide-killed samples, showed no significant differences compared to the ground sample, suggesting a comparable level chlorophyll levels across these conditions. A slight variation in the I_1549/1359_ found in FL-Type II could potentially be due to the variation in chlorophyll *a* to chlorophyll *b* ratio as vibration corresponds to the stretching vibrations of the carbonyl (C=O) group in the aldehyde group present in chlorophyll *b* also contributes to the intensity of Raman vibration at 1549 cm^− 1^ (this ratio has slightly increased in FL-Type II samples). In addition, a minor shift in the 1549 cm^− 1^ band to 1546 cm^− 1^ in FL-Type II samples was also found, which could possibly be attributed to the change in the electronic environment around the chlorophyll molecules as an adaptive response to radiation stress and subsequent variation in chlorophyll *b* content.

## Discussion

### Artemis-1

The effects of the galactic cosmic environment on biological samples remains largely unknown, as the samples returned on Artemis-1 were the first samples returned following exposure to galactic cosmic environment beyond low Earth orbit since the Apollo era.

### Viability shift: protection from cosmic radiation

The major finding of this study is that our hypothesis that exposure to the galactic cosmic environment would reduce *Chlamydomonas* survival through the process of necrosis was disproven. The alternative balancing argument that the radiation stimulus would induce a population survival response via a PCD pathway is supported. These findings are initially surprising, as the galactic cosmic environment contains high linear energy transfer (LET) particles that evoke complex DNA and other cellular damage^27,28^, including significant damage to biological organisms, that may include space radiation-induced carcinogenesis, cardiovascular disease, and central nervous system deficiencies^29,30^. Approximately 70% of galactic cosmic particles are high energy protons with similar relative biological effectiveness (RBE) slightly higher than low LET radiation such as gamma rays and X-rays. Protons are passing through living organisms with uniform distributions. However, heavier particles, which can cause devastating consequences on cells with direct hits, are distributed non-uniformly through the cell populations. Secondary effects are then caused by secondary particles or by signal transduced from the cells with direct hits^25–28^.

The findings can be explained by understanding the properties of PCD^10,11,13,31^. PCD is a population stress response that is both adaptive and plastic^11^. Initial population decline due to PCD in response to stress helps the population rebound^11^. PCD can be a group-level stress response, where there is reconstitution of population density by expansion of survivors^31^. Microgravity simulation has been found to induce PCD in multiple cells, and tissues, both in vivo and in vitro^27^.

Importantly, although the apoptotic shift in *Chlamydomonas* following exposure to cosmic radiation is counterintuitive, doses of diverse forms of ionizing radiation, similar to the space exposure of our samples, have previously been shown to stimulate growth and raised suggested mechanisms for radiation-induced changes^32–34^. Kim et al.^32^ explored the counterintuitive bioeffects of ionizing radiation, such as X- and γ-rays, and their efficacy as an elicitor to facilitate batch or fed-batch cultivation of *Chlamydomonas* cells. A certain dose range of X- and γ-rays, similar to the exposure of our samples in space, was shown to stimulate the growth and metabolite production of *Chlamydomonas* cells, substantially increasing chlorophyll, protein, starch, and lipid content as well as growth and photosynthetic activity. Kim et al.^32^ postulate that these findings may be explained by epigenetic stress memory or priming effects associated with ROS-mediated metabolic remodeling. Giardi et al.^33^ flew *Chlamydomonas* algal mutants in low Earth orbit. The mutants contained specific amino acid substitutions in the functionally important regions of the pivotal Photosystem II (PSII) reaction center D1 protein near the QB binding pocket and in the environment surrounding Tyr-161 (YZ) electron acceptor of the oxygen-evolving complex. Two D1 mutants, I163N and A251C, performed efficient photosynthesis, and actively re-grew upon return to Earth. Mimicking the neutron irradiation component of cosmic rays on Earth yielded similar results^33^. These results highlighted the contribution of D1 conformation in relation to photosynthesis and oxygen production in space. When *C. reinhardtii* was grown exposed to chronic radiation from a Cesium-137 source, there was a significant increase in biomass accumulation under lunar radiation level compared to LEO or Earth radiation levels, demonstrating that ionizing radiation can affect growth of *C. reinhardtii*^34^.

There are many methods to assay PCD in algae and specifically in *Chlamydomonas reinhardtii*^12^. PCD and necrosis assayed by flow cytometry analysis of Annexin V binding and propidium iodide uptake (described as a ‘hard sign’ of PCD), or cell size, give the best specificity and sensitivity of methods available within the parameters of the Artemis-1 mission profile^35^. Multiparameter flow cytometry can provide more robust data analysis^36^, but no matter which combination of parameters we compared, there was always more PCD, and less necrosis in the spaceflight samples compared to the ground controls.

### Insertion of the tardigrade gene *Dsup*: protection from cosmic radiation

Amongst the tardigrade intrinsically disordered proteins^37^, *Dsup* was our choice to insert into the nucleus of *Chlamydomonas* based on evidence that Dsup suppresses the occurrence of DNA breaks by radiation in human-cultured cells^6^. Inserting *Dsup* into the nucleus of *Chlamydomonas* reduced necrosis, both in space and in the ground-based control samples. The starting baseline necrosis in the ground and flight samples were very different. Certainly, Dsup induced a smaller decrease in necrosis in space than on the ground, but it is unclear whether this is a ceiling phenomenon, as the Dsup-induced final survival level is no different in the flight and ground groups.

### Raman spectroscopy and redox metabolism during spaceflight

Redox signaling is activated in diverse tissues and cell systems by varied forms of stress, including radiation, spaceflight, and simulated microgravity^38,39^. Stress-induced redox activation can be alternatively adaptive or contribute to pathological outcomes^39^. Targeting of redox metabolism has been proposed for mitigation of radiation injury^40^. Carotenoids are a class of pigments critical for photoprotection^41^. It is likely that cosmic ray and/or microgravity-induced stress in *Chlamydomonas* could potentially activate their defense mechanisms to prevent radiation damage, which can result in enhanced carotenoid biosynthesis and production of carotenoids^42^ along with other defense mechanisms. β-carotene administration is the subject of multiple clinical trials aiming to decrease cardiovascular disease or cancer risk^43^.

The differential activation of unique survival strategies under space conditions, as observed through Raman spectroscopy in *Chlamydomonas*, highlights the complex nature of cellular responses to environmental stresses such as radiation and microgravity. The Raman spectroscopic analyses detected two types of cells, which we have designated as Type I and Type II. The enhanced production of carotenoids seen in Type II cells from the flown sample served as one of the protective mechanisms, likely activated to scavenge reactive oxygen species generated by stress, thereby mitigating potential radiation damage. This aligns with various studies suggesting that carotenoid biosynthesis can be stimulated as an adaptive response to environmental challenges^44,45^. We also saw some algal cells with weak carotenoid Raman bands (Type I) in the same flight samples, which is consistent with the flow cytometry and fluorescence microscopy data presented in this manuscript, that not all the cells can survive the cosmic ray stress.

The reduction of significant carotenoid vibrations in Type I cells (also in the azide-killed samples) suggests that these cells may not effectively induce protective responses compared to carotenoid-rich cells, making them more susceptible to radiation-induced damage. This observation is critical as it implies that not all cells within a population will uniformly respond to stress, highlighting the importance of understanding individual cellular adaptations in space biology. In addition, our findings also underline the utility of Raman spectroscopy in detecting subtle changes in cellular composition and stress responses, such as shifts in carotenoid ratios and alterations in the electronic environment around chlorophyll molecules. These insights are pivotal for developing strategies to enhance the resilience of microorganisms in space.

The European Space Agency (ESA) attached an external platform, known as EXPOSE, to the outside the Russian Zvezda module, that is part of the International Space Station^46^. EXPOSE allowed exposure of chemical and biological samples to the vacuum and radiation conditions outside the ISS inside the radiation protective Van Allen belts^7^.

The EXPOSE-R2 series of experiments included two biological payloads, namely the BIOlogy and Mars Experiment (BIOMEX)^17^ and the Biofilm Organisms Surfing Space (BOSS)^47^. The EXPOSE-R2 series of experiments helps puts the current data in perspective, as both projects analyzed samples returned from the ISS by Raman spectroscopy.

The BIOlogy and Mars Experiment (BIOMEX) investigated extremophile endurance and biosignature detectability when mixed with Mars or Moon regolith simulants^17,18^. Desiccated cells of two carotenoids containing organisms, the cyanobacterium *Nostoc sp*. and the green alga *Sphaerocystis sp*. were exposed to space and simulated Mars-like conditions in space in the presence of two Martian mineral analogues and a Lunar regolith analogue. Carotenoids in both organisms were still detectable at relatively high levels after being exposed for 15 months in Low Earth Orbit to UV, cosmic rays, vacuum (or Mars-like atmosphere) and temperatures stresses in all sample types^17,18^.

The BOSS (Biofilm Organisms Surfing Space) experiment showed that dried biofilms, that is microorganisms embedded in extracellular polymeric substances, better faced long-term permanence in space than dried planktonic counterparts^47^.

### Cost and practicality of moonshot hardware

Advances in materials, microelectronics, and molecular and cellular biology technologies provided a firm basis to produce cost-effective customized spaceflight hardware. Bionetics Corporation’s engineering team developed new hardware, called Moonshot, within a cost that could be supported from the funded grant. Moonshot holds three 10-cm diameter segmented Petri dishes, and provides timed daily blue and red-light sample exposure, while recording state, battery voltage, temperature, and acceleration in three dimensions at programmable times. The Moonshot hardware performed flawlessly, providing flight certified, flight proven hardware for future biological investigations^20^.

### Radiation dose

The two sensors adjacent to the Moonshot hardware on Artemis I had different radiation spectrum sensitivities^8^. The CAD was expected to have a slightly higher reading that the RAM, due to differences in radiation spectrum collection by the two dosimeters^8^. Solar energetic particles from solar flares or sunspots can moderate the observed radiation levels but no solar flares or sunspots occurred during the Artemis I mission. Two ‘phantoms’ of the Matroshka AstroRad Radiation Experiment (MARE) flew in two passenger seats in the Orion capsule and allowed detailed definition of the linear energy transfer spectrum the samples were exposed to^48,49^.

The levels of radiation recorded in the RAM and CAD detectors during the Artemis-1 flight are similar to levels seen during the Curiosity rover flight to Mars, if the same period of time is extracted from the total radiation levels reported^50^. The radiation levels during the Artemis-1 mission flight were far greater than ambient terrestrial levels^51^, about three times the levels on the ISS^7^, and about the same total exposure as a medical computed tomography (CT) abdominal scan with and without contrast (Table 1)^52,53^. CAD and RAM measure quantity, but do not define the spectrum of the linear energy transfer for the galactic cosmic environment^8^. The radiation risk is directly influenced by this spectrum as the quality of the radiation characterized by its pattern of energy deposition at the micron/DNA scale determines damage^28^. The linear energy transfer profile as the Curiosity rover flew to Mars has been documented^50^. The linear energy transfer spectrum for the Artemis-1 flight has been defined^48^, but a basis for biological interpretation is lacking.

### Parsing the elements of the galactic cosmic environment

Ideally, we would model and study the elements of microgravity, convection, and radiation of the galactic cosmic environment individually in ground-based studies. Unfortunately, the technical expertise to parse the elements of the galactic cosmic environment are lacking. Microgravity simulations balance gravity with an induced equal and opposite force, typically using liquid culture environment^54^. These model systems introduce multiple new stimuli, limiting comparison to an element of the galactic cosmic environment. There are scant, if any, mechanisms to model biological responses to low convection^54^. NASA’s ground-based Galactic Cosmic Ray Simulator at the NASA Space Radiation Lab^55^ will be critical to understand the biology of galactic cosmic radiation but is beyond the scope and resources of the current study.

### Benefits and limitations

The galactic cosmic environment poses many challenges but also provides opportunities. For life to survive in cosmic radiation, the first steps begin with research to understand the fundamental effect on biological processes; understand the fundamental biology of life during exposure to cosmic radiation; contribute knowledge to reduce human health risk beyond low Earth orbit; and contribute to improved system performance and reduced system risk. This data set is a first step to answer fundamental questions such as can we survive and thrive beyond Earth, and can we use knowledge gained from studying the biological effects of galactic space radiation for Earth-based benefits?

## Conclusions

Within the limitations of the physical handling of specimens necessitated by the Artemis-1 mission, flight around the Moon with galactic cosmic environment exposure allows multiple conclusions. Some *Chlamydomonas* remained viable and grew robustly on return to Earth. Flown samples exposed to the galactic cosmic environment had increased programmed cell death and decreased necrosis compared to ground controls. Insertion of the *Dsup* tardigrade gene was protective both on the ground and in flight, although the ground effect was far larger numerically. Raman spectroscopy analysis showed that a redox-protective terpenoid carotene pigments, known cell death mediators, were increased during flight around the Moon, defining this technology as an important new non-destructive tool for analysis of biological space flight samples. An inexpensive simple new flight hardware, termed Moonshot, can perform flawlessly, and is available as flight-certified, flight-proven hardware for timed illumination and monitoring of samples for flight and terrestrial applications.

## Methods and materials

### Chemicals and plasticware

Chemicals and plasticware utilized with catalogue numbers and source include: Celltreat RNAase and DNAase free Polysytrene 16 ml four-compartment Petri dishes 229684X Celltreat Pepperell MA; Propidium iodide 1 mg/ml in water P4864 from Millipore Sigma St. Louis MO; Flowmi cell strainer, 40 μm porosity 136,800,040 SP Bel-Art South Wayne, NJ; 96 Well Conical V Bottom Plate 12-567-209 and MAX Efficiency Transformation Reagent A24229 from Thermo Scientific Waltham MA; Phosphate buffered saline P4417 Sigma Aldrich St. Louis MA; and from Life Technologies Eugene OR, Annexin- V Alexa Fluor 488 conjugate A13201, and Annexin binding buffer V13246. All other chemicals were purchased from Sigma/Aldrich, now Millipore-Sigma (Burlington, MA).

### Chlamydomonas reinhardtii

*Chlamydomonas reinhardtii* CC-4533 background strain was purchased from the University of Minnesota Chlamydomonas collection (Minneapolis, MN).

### *Chlamydomonas reinhardtii Dsup* transformant

The *Chlamydomonas* were spotted on Tris-acetate-phosphate (TAP) agar^56^ on polystyrene plates, and aliquots reseeded onto fresh plates robotically every 2 weeks, as described^57^. The Intronserter tool^58^ was employed to optimize the codon usage of the *Dsup* protein and to incorporate the necessary introns for efficient expression in *C. reinhardtii.* The modified gene block was synthesized by Twist Bioscience. The codon-optimized *Dsup* gene was fused to an mRuby2 fluorescent protein tag at the N-terminus to enable expression analysis.

Transformation of *Chlamydomonas reinhardtii* was performed using electroporation. Cells were cultured in TAP medium under standard conditions until reaching a density of 1–2 million cells/mL and harvested by centrifugation at 6000 rpm for 5 min. The cell pellet was washed twice with MAX Efficiency Transformation Reagent and resuspended to a final concentration of 200–300 million cells/mL. Linearized plasmid DNA (~ 4 µg) was mixed with 250 µL of the cell suspension and incubated at 4 °C for 5 min. Electroporation was conducted in a 0.4-cm at 500 V, 50 µF, and 800 Ω, with a pulse duration of approximately 30 ms. Following electroporation, cells were allowed to recover at room temperature for 15 min before being transferred to TAP-40 mM sucrose solution and incubated for 14–16 h. Next, cells were subsequently centrifuged, resuspended in TAP medium, and plated on selective TAP-agar plates. Plates were incubated in an algal chamber for 5–7 days to allow colony formation. 8 individual clones were isolated as single colonies.

The *Dsup* insert was validated by sequencing to confirm the integrity of the inserted sequence; protein localization through fluorescence microscopy of the tagged Dsup protein; and gene insertion through PCR, to verify the presence of the *Dsup* gene in the *Chlamydomonas* genome.

### Equipment

Flow cytometry was performed on a Becton-Dickinson Accuri C + Plus flow cytometer (Franklin Lakes, NJ). Raman spectra were collected using a Horiba LabRAM Odyssey Raman microscope equipped with a 785 nm diode laser.

Moonshot hardware was designed and built by The Bionetics Corporation, (Kennedy Space Center, Brevard County, FL) as an ultralow-power growth system for use on Artemis-1^20^. Each of the three growth modules holds one standard 100 mm diameter circular Petri dish. The plates are illuminated with blue (∼450 nm) and red (∼660 nm) light-emitting diode (LED) lights continuously for 6 h in every 24 h. The combination of red and blue light is optimum for *Chlamydomonas*^59,60^. The hardware monitors and records the temperature of the experiment, the power level, and draw from the batteries (as an indication that the lights were turned on and off), as well as the acceleration levels in three axes.

Once activated and loaded with the biological samples, the flight hardware was placed in a custom lathed form-fitting Styrofoam support and encased in a Nitex bag. The assembly was termed the BioExpt-01 Science Bag.

Crew Active Dosimeter (CAD) and Radiation Area Monitor (RAM) radiation detectors were placed adjacent to the biological flight hardware in the BioExpt-01 Science Bag. The detectors had been assembled and delivered to NASA/Kennedy Space Center prior to flight by the Space Radiation Analysis Group based at NASA Johnson Space Center (JSC) by Human Health and Performance Directorate/Leidos personnel. The detectors were collected post-flight and data was analyzed at NASA JSC^8^.

### Preflight sample handling

A stab of *Chlamydomonas reinhardtii* CC-125 was received from the Chlamydomonas Resource Center and aliquots inoculated into 5 ml TAP with a sterile loop. Once initial growth was observed by visual color change, and observation of swimmers by epifluorescent microscopy, TAP media was added to 100 ml final volume and pools were grown at room temperature for 4 days with 12 h of light/dark low light (~ 5 µmol photons m-2 s-1). Samples were pinned using a 1536 pin tool with pins removed to fit into 3 of the 4 quadrants of the round 10 cm diameter 4 quadrant plates. The 4th quadrant was hand spotted with *Dsup* and azide-killed samples. After pinning, cells were allowed to grow at room temp ~ 20^o^C for 48 h with 12 h of light/dark. Plates were stored at 4 ^o^C for 5 days due to a launch delay, then warmed to room temperature and transported to Kennedy Space Center for integration into the Moonshot flight hardware, then handover of the hardware to NASA flight engineers for installation in the Orion capsule.

Three sets of three plates of *Chlamydomonas* samples were spotted in the Nislow lab in the Faculty of Pharmaceutical Sciences at the University of British Columbia, and shipped overnight to Bionetics Corporation in the Space Life Sciences Building at Kennedy Space Center. Two sets of spotted plates were loaded into a pair of identical flight-certified Moonshot space flight hardware sets^20^. One hardware set was placed in the Orion capsule on the Space Launch System and flew on Artemis I. The identical twin to the flight set was held at the Bionetics facility and the expected temperature profile mimicked daily.

The third set of spotted plates was hand carried by air (without radiation) to the parent lab in the Durham VA Health Care System in Durham NC, and placed in a prototype of the flight hardware, with the same capabilities of the flight hardware, but not in a bonded setting to maintain flight certification. This control set was monitored three times per week with confirmation of the presence of swimming alga by light microscopy of the smear of a spot on a slide, biweekly confirmation of viable alga by inoculation into TAP media and observing growth, and weekly confirmation of viable alga by flow cytometry exclusion of propidium iodide.

### Artemis-1 flight

After an initial delay of 19 days on the launch pad, the Artemis − 1 mission was launched from Kennedy Space Center (KSC) on November 16, 2022 for a planned 25.5-day space mission. Orion completed one flyby of the Moon on November 21, followed by a distant retrograde orbit for six days and then a second flyby of the Moon on November 25, and subsequently returned to Earth.

### Post flight sample handling

The Orion capsule was recovered from the Pacific Ocean, returned to California, and transported back to KSC by truck. However, the Space Biology experiments were first removed from Orion capsule in California, flown on an accompanied commercial flight to Kennedy Space Center at ambient temperature (without security radiation scanning) and the Moonshot hardware containing the samples, released to the investigators 9 days after the landing. The sample arrived back in the investigator’s lab three days later, after decommissioning of the hardware at Bionetics facility adjacent to KSC. The samples for the payload described in the report were in the hardware for a total of 56.5 days (Fig. 1). The flight samples were exposed to microgravity in space, but also gravity transitions during launch and re-entry.

Samples from 3 quadrants of the three flight and ground plates were washed off the plates by flooding with 5 ml TAP, pelleting by centrifugation, and aliquoted for various analyses. The hand spotted *Dsup* and azide-killed samples were lifted off individually with a sterile loop, placed in 1 ml TAP, and aliquoted for analysis.

### Flow cytometry

Flow cytometry analysis assayed propidium iodide and annexin staining^61^. Propidium Iodide is a fluorescent nucleic acid dye used as a viability marker, which binds only to double-stranded nucleic acids, and, as it is non membrane permeable, it can only bind to DNA in dead cells that have compromised membranes. Annexin V is a protein that binds to phosphatidylserine (PS), a marker of apoptosis, and is used to detect apoptotic cells in flow cytometry when fluorescently conjugated.

In preliminary experiments, both PI and Alexa Fluor 488 were titrated against *Chlamydomonas* on a standard curve of dilutions to find peak binding, with greater than one log shift in specific signal strength versus unstained controls by flow cytometry. Six colonies from flight and ground plates, plus four *Dsup* colonies from flight and ground plates, were harvested and resuspended in annexin-binding buffer (10 mM HEPES, 140 mM NaCl, 2.5 mM CaCl2 pH 7.4), The aggregates were disrupted by vortexing and then filtered through a 40 μm nylon cell strainer, to remove clumps that would plug the flow cytometer. The resuspended *Chlamydomonas* samples were aliquoted 100 µl per well into V-bottom microtiter plates. Individual aliquots were either stained with Alexa Fluor 488-annexin (5 µl/well) and propidium iodide (PI) (1 µl/well of 1 mg/ml in water) to identify PCD and necrosis.

We counted 2,000 *Chlamydomonas* cells from each sample. Background signal was estimated by appropriate no-dye controls. In every flow cytometry run, quality controls were performed on the instrument with three-color fluorescent beads, followed by assay of five control tubes: (1) no dyes, (2) Alexa fluor 488 annexin binding alone, (3) propidium iodide alone, and (4) Alexa fluor 488 annexin binding with propidium iodide.

### Single-cell confocal Raman spectroscopy of *Chlamydomonas reinhardtii*

Multiple colonies of *Chlamydomonas* were harvested from a ground plate, and flight plate. The samples included colonies flown live and colonies pretreated with azide before flight. The colonies were harvested into 1.5 ml Eppendorf tubes with 500 µL TAP and resuspended by pipetting and vortexing. The resultant *Chlamydomonas* solution was filtered through a 40 μm mesh into 12 × 74 mm polypropylene tubes to remove multicellular aggregates. The remaining solution of cells was divided into two 500 µL Eppendorf tubes, spun at 3000 g for 1 min to pellet the cells, and the supernatant aspirated. One tube was left to air dry. The second tube had 100 µL of 10% electron microscopy paraformaldehyde added, incubated for 10 min, and then was spun to pellet the cells before the supernatant was aspirated. This paraformaldehyde-fixed sample was washed with 500 µL deionized water, spun, and the supernatant aspirated.

A 2 µl aliquot of each sample rehydrated with distilled water was spotted on a quartz slide (25 mm x 25 mm x 1 mm) that was kept on a microscopic glass slide covered with aluminum foil. This substrate showed minimal background signal compared to various other substrates we tried such as glass, CaF_2_ and Al_2_O_3_. A silicon wafer was not used as it has a strong Raman band at 521 cm^− 1^ and interfered with the current analysis. The samples were air-dried at room temperature and were analyzed. A Horiba LabRAM Odyssey Raman microscope (spectral resolution of ≤ 0.2 cm⁻¹ (FWHM) at 785 nm excitation) was used for the Raman spectral characterization of these samples. Laser power density was optimized (surface laser power at ~ 8 mW using a 100X objective) in order to achieve better spectral intensity with characteristic Raman bands, which were not present at lower laser power densities. Exposure time and laser intensity were optimized by conducting a series of experiments to prevent charring of the samples to eliminate any unwanted signals due to laser-induced biomolecule denaturation. A 785 nm laser was used to acquire Raman spectra (acquisition time of 30 s). First, white light focused on the individual algal cells at a magnification of 100X. Then, Raman spectra were collected in the range of 400–1800 cm^− 1^. The spectral baselines were pre-processed by polynomial fitting.

### Statistics

Flow cytometric data are presented as geometric mean ± standard error with six replicates (unless otherwise noted). Flow cytometric data were evaluated by two-tailed Student’s t-test comparing flown versus ground control *Chlamydomonas* using Statistica 6.1 (StatSoft Inc. Tulsa OK).

For the Raman spectral analysis: Spectra were collected from five or more algal samples across three different colonies exposed to various conditions and subsequently averaged to better represent statistical variations in the intensities of vibrations corresponding to various biomolecular components within the spectra. The standard deviation of these intensities under different conditions was illustrated using error bars in the histogram to provide a clear visualization of the variability and reliability of our measurements.

## Data Availability

Contact Tim Hammond, the corresponding author, for information.

## References

[1] Bokharia, R. S. et al. Looking on the horizon; potential and unique approaches to developing radiation countermeasures for deep space travel. *Sci. Direct*. **35**, 105–112 (2022). 10.1016/j.lssr.2022.08.003 36336356

[2] Torzillo, G., Scoma, A., Faraloni, C. & Giannelli, L. Advances in the biotechnology of hydrogen production with the microalga Chlamydomonas reinhardtii. *Crit. Rev. Biotechnol.* **35** (4), 485–496 (2015). 24754449 10.3109/07388551.2014.900734

[3] Scaife, M. A. et al. Establishing Chlamydomonas reinhardtii as an industrial biotechnology host. *Plant. J.* **82** (3), 532–546 (2015). 25641561 10.1111/tpj.12781 PMC4515103

[4] Tavakol, D. N. et al. Modeling and countering the effects of cosmic radiation using bioengineered human tissues. *Biomaterials* **301**, 122267 (2023). 37633022 10.1016/j.biomaterials.2023.122267 PMC10528250

[5] Puig, J., Knodlseder, N., Quera, J., Algara, M. & Guell, M. DNA damage protection for enhanced bacterial survival under simulated low Earth orbit environmental eonditions in *Escherichia coli*. *Front. Microbiol.* **12**, 789668 (2021). 34970246 10.3389/fmicb.2021.789668 PMC8713957

[6] Hashimoto, T. & Kunieda, T. DNA protection protein, a novel mechanism of radiation tolerance: lessons from tardigrades. *Life (Basel)*. **7**, 26. 10.3390/life7020026 (2017). 28617314 10.3390/life7020026 PMC5492148

[7] Kodaira, S. et al. Space radiation dosimetry at the exposure facility of the international space station for the Tanpopo mission. *Astrobiology* **21** (12), 1473–1478 (2021). 34348047 10.1089/ast.2020.2427

[8] Gaza, R. et al. The importance of time-resolved personal dosimetry in space: the ISS crew active dosimeter. *Life Sci. Space Res.* **39**, 95–105 (2023). 10.1016/j.lssr.2023.08.004 37945094

[9] Bertalan, I., Esposito, D. & Torzillo, G. Photosystem II stress tolerance in the unicellular green Alga Chlamydomonas Reinhardtii under space conditions. *Microgravity Sci. Technol.* **19**, 122–127 (2007).

[10] Durand, P. M., Choudhury, R., Rashidi, A. & Michod, R. E. Programmed death in a unicellular organism has species-specific fitness effects. *Biol. Lett.* **10**, 20131088. 10.1098/rsbl.2013.1088 (2014). 24573154 10.1098/rsbl.2013.1088 PMC3949379

[11] Zeballos, N., Grulois, D., Leung, C. & Chevin, L. M. Acceptable loss: fitness consequences of salinity-induced cell death in a halotolerant microalga. *Am. Nat.* **201** (6), 825–840 (2023). 37229704 10.1086/724417

[12] Barreto Filho, M. M., Bagatini, I. L. & Durand, P. M. How shall we measure programmed cell death in eukaryotic microalgae? *Eur. J. Phycol.* **58** (1), 13–34 (2022).

[13] Durand, P. M. & Ramsey, G. The concepts and origins of cell mortality. *Hist. Phil. Life Sci.* **45**, 23 (2023). 10.1007/s40656-023-00581-8 10.1007/s40656-023-00581-8 PMC10250477 37289372

[14] Panikkanvalappil, S. R., Hira, S. M., Mahmoud, M. A. & El-Sayed, M. A. Unraveling the biomolecular snapshots of mitosis in healthy and cancer cells using plasmonically-enhanced Raman spectroscopy. *J. Am. Chem. Soc.* **136** (45), 15961–15968 (2014). 25330058 10.1021/ja506289u PMC4235372

[15] Panikkanvalappil, S. R., Hira, S. M. & El-Sayed, M. A. Elucidation of ultraviolet radiation-induced cell responses and intracellular biomolecular dynamics in mammalian cells using surface-enhanced Raman spectroscopy. *Chem. Sci.* **7** (2), 1133–1141 (2016). 29910869 10.1039/c5sc03817k PMC5975792

[16] Sharma, S. et al. Diverse organic-mineral associations in Jezero crater. *Mars Nature*. **619**, 724–732 (2023). 37438522 10.1038/s41586-023-06143-z PMC10371864

[17] de Vera, J. P. et al. Limits of life and the habitability of mars: the ESA space experiment BIOMEX on the ISS. *Astrobiology* **19**, 145–157 (2019). 30742496 10.1089/ast.2018.1897 PMC6383581

[18] Baque, M. et al. Biosignature stability in space enables their use for life detection on Mars. *Sci. Adv.* **8** (36), eabn7412 (2022). 36070383 10.1126/sciadv.abn7412 PMC9451166

[19] Panikkanvalappil, S. R. et al. Hyperoxia induces intracellular acidification in neonatal mouse lung fibroblasts: Real-Time investigation using plasmonically enhanced Raman spectroscopy. *J. Am. Chem. Soc.* **138** (11), 3779–3788 (2016). 26938952 10.1021/jacs.5b13177

[20] Hammond, T. G. et al. Moonshot: affordable, simple, flight hardware for the Artemis-1 mission and beyond. *Front. Space Technol.* **1**, 593523. 10.3389/frspt.2020.593523 (2020).

[21] Jehlička, J. et al. Potential and limits of Raman spectroscopy for carotenoid detection in microorganisms: implications for astrobiology. *Philosophical Trans. Royal Soc. A: Math. Phys. Eng. Sci.* **372** (2030), 20140199 (2014). 10.1098/rsta.2014.0199 PMC4223861 25368348

[22] Udensi, J., Loughman, J., Loskutova, E. & Byrne, H. J. Raman spectroscopy of carotenoid compounds for clinical Applications—a review. *Molecules* **27**10.3390/molecules27249017 (2022). 10.3390/molecules27249017 PMC9784873 36558154

[23] de Oliveira, V. E., Castro, H. V., Edwards, H. G. M. & de Oliveira, L. F. C. Carotenes and carotenoids in natural biological samples: a Raman spectroscopic analysis. *J. Raman Spectrosc.* **41** (6), 642–650 (2010).

[24] Udensi, J., Loughman, J., Loskutova, E. & Byrne, H. J. Raman spectroscopy of carotenoid compounds for clinical applications-A review. *Molecules* **27**, 9017. 10.3390/molecules27249017 (2022). 36558154 10.3390/molecules27249017 PMC9784873

[25] Vítek, P., Osterrothová, K. & Jehlička, J. Beta-carotene—A possible biomarker in the Martian evaporitic environment: Raman micro-spectroscopic study. *Planet. Space Sci.* **57** (4), 454–459 (2009).

[26] Cai, Z. L., Zeng, H., Chen, M. & Larkum, A. W. D. Raman spectroscopy of chlorophyll d from acaryochloris marina. *Biochim. Et Biophys. Acta (BBA) - Bioenergetics*. **1556** (2), 89–91 (2002). 10.1016/s0005-2728(02)00357-2 12460664

[27] Prasad, B. et al. Influence of microgravity on apoptosis in cells, tissues, and other systems in vivo and in vitro. *Int. J. Mol. Sci.* **21**, 9373. 10.3390/ijms21249373 (2020). 33317046 10.3390/ijms21249373 PMC7764784

[28] Papadopoulos, A. et al. Space radiation quality factor for galactic cosmic rays and typical space mission scenarios using a microdosimetric approach. *Radiat. Environ. Biophys.* **62** (2), 221–234 (2023). 37062024 10.1007/s00411-023-01023-6 PMC10188414

[29] Mehner, C. et al. Real versus simulated galactic cosmic radiation for investigating cancer risk in the hematopoietic system - are we comparing apples to apples? *Life Sci. Space Res.* **29**, 8–14 (2021). 10.1016/j.lssr.2021.01.001 33888292

[30] Sishc, B. J. et al. The need for biological countermeasures to mitigate the risk of space radiation-Induced carcinogenesis, cardiovascular disease, and central nervous system deficiencies. *Life Sci. Space Res.* **35**, 4–8 (2022). 10.1016/j.lssr.2022.06.003 36336368

[31] Kojic, M. & Milisavljevic, M. When disaster strikes: reconstitution of population density by expansion of survivors. *Mol. Ecol.* **29** (24), 4757–4764 (2020). 33047408 10.1111/mec.15680

[32] Kim, J. H. et al. Application of ionizing radiation as an elicitor to enhance the growth and metabolic activities in Chlamydomonas reinhardtii. *Front. Plant. Sci.* **14**, 1087070 (2023). 36890890 10.3389/fpls.2023.1087070 PMC9986495

[33] Giardi, M. T. et al. Mutations of photosystem II D1 protein that empower efficient phenotypes of Chlamydomonas reinhardtii under extreme environment in space. *PloS One*. **8** (5), e64352 (2013). 23691201 10.1371/journal.pone.0064352 PMC3653854

[34] Hersh, H. L., Hannah, L. C. & Settles, A. M. *Determining Chlamydomonas reinhardtii resistance to ionizing radiation at a genome-wide scale*. In *37th Annual Meeting of the American Society for Gravitational and Space Research*. Baltimore, MD, USA. (2021).

[35] Durand, P. M. Mechanisms and measures of programmed cell death in the unicellular world. In *The Evolutionary Origins of Life and Death* 89–105. (University of Chicago Press, 2021).

[36] Ding, M. & Baker, D. Recent advances in high-throughput flow cytometry for drug discovery. *Expert Opin. Drug Discov*. **16** (3), 303–317 (2021). 33054417 10.1080/17460441.2021.1826433

[37] Kasianchuk, N., Rzymski, P. & Kaczmarek, L. The biomedical potential of tardigrade proteins: A review. *Biomed. Pharmacother*. **158**, 114063. 10.1016/j.biopha.2022.114063 (2023). 36495665 10.1016/j.biopha.2022.114063

[38] Kamal, K. Y. & Lawler, J. M. Cellular and molecular signaling Meet the space environment. *Int. J. Mol. Sci.* **24**, 5955. 10.3390/ijms24065955 (2023). 36983029 10.3390/ijms24065955 PMC10058013

[39] Pohjoismaki, J. L. O. & Goffart, S. Adaptive and pathological outcomes of radiation stress-induced redox signaling. *Antioxid. Redox. Signal.* **37** (4–6), 336–348 (2022). 35044250 10.1089/ars.2021.0257

[40] Farhood, B. et al. Targeting of cellular redox metabolism for mitigation of radiation injury. *Life Sci.* **250**, 117570. 10.1016/j.lfs.2020.117570 (2020). 32205088 10.1016/j.lfs.2020.117570

[41] Caferri, R., Guardini, Z., Bassi, R. & Dall’Osto, L. Chapter Two—Assessing photoprotective functions of carotenoids in photosynthetic systems of plants and green algae. In *Methods in Enzymology*, (eds. E.T. Wurtzel) 53–84. (Academic Press, 2022). 10.1016/bs.mie.2022.04.006 36008020

[42] Bast, A., van der Plas, R. M., van den Berg, H. & Haenen, G. R. Beta-carotene as antioxidant. *Eur. J. Clin. Nutr.* **50**, S54–56 (1996). 8841774

[43] Patrick, L. Beta-carotene: the controversy continues. *Altern. Med. Rev.* **5** (6), 530–545 (2000). 11134976

[44] Sankari, M., Hridya, H., Sneha, P., George Priya Doss, C. & Ramamoorthy, S. Effect of UV radiation and its implications on carotenoid pathway in Bixa orellana L. *J. Photochem. Photobiol B*. **176**, 136–144. 10.1016/j.jphotobiol.2017.10.002 (2017). 28992607 10.1016/j.jphotobiol.2017.10.002

[45] Saini, R. K. & Keum, Y. S. Significance of genetic, environmental, and Pre- and postharvest factors affecting carotenoid contents in crops: A review. *J. Agric. Food Chem.* **66** (21), 5310–5324 (2018). 29745660 10.1021/acs.jafc.8b01613

[46] Rabbow, E. et al. EXPOSE, an Astrobiological exposure facility on the international space station - from proposal to flight. *Orig Life Evol. Biosph*. **39** (6), 581–598 (2009). 19629743 10.1007/s11084-009-9173-6

[47] Cottin, H., & Rettberg, P. EXPOSE-R2 on the International Space Station. (2014–2016): results from the PSS and BOSS Astrobiology Experiments. *Astrobiology* **19**(8), 975–978 (2019). 10.1089/ast.2019.0625 31373529

[48] George, S. P. et al. Space radiation measurements during the Artemis I lunar mission. *Nature* **634** (8032), 48–52 (2024). 39294379 10.1038/s41586-024-07927-7 PMC11446838

[49] Berger, T. et al. The German aerospace center M-42 radiation detector-A new development for applications in mixed radiation fields. *Rev. Sci. Instrum.* **90**, 125115. 10.1063/1.5122301 (2019). 31893784 10.1063/1.5122301

[50] Zeitlin, C. et al. Measurements of energetic particle radiation in transit to Mars on the Mars science laboratory. *Science* **340** (6136), 1080–1084 (2013). 23723233 10.1126/science.1235989

[51] Ruhm, W. et al. Typical doses and dose rates in studies pertinent to radiation risk inference at low doses and low dose rates. *J. Radiat. Res.* **59** (suppl_2), ii1–ii10 (2018). 29432579 10.1093/jrr/rrx093 PMC5941142

[52] *Radiation Dose from X-rays and CT Exams* Available from: https://www.radiologyinfo.org/en/info/safety-xray

[53] Buchberger, B., Scholl, K., Krabbe, L., Spiller, L. & Lux, B. Radiation exposure by medical X-ray applications. *Ger. Med. Sci.* **20**, Doc06 (2022). 35465642 10.3205/000308 PMC9006309

[54] Hammond, T. G. & Hammond, J. M. Optimized suspension culture: the rotating-wall vessel. *Am. J. Physiol. Ren. Physiol.* **281** (1), F12–25 (2001). 10.1152/ajprenal.2001.281.1.F12 11399642

[55] Simonsen, L. C., Slaba, T. C., Guida, P. & Rusek, A. NASA’s first ground-based galactic cosmic ray simulator: enabling a new era in space radiobiology research. *PLoS Biol.* **18**, e3000669. 10.1371/journal.pbio.3000669 (2020). 32428004 10.1371/journal.pbio.3000669 PMC7236977

[56] Mitra, M. et al. Isolation and characterization of a novel bacterial strain from a Tris-Acetate-Phosphate agar medium plate of the green micro-alga Chlamydomonas reinhardtii that can utilize common environmental pollutants as a carbon source. *F1000Res* **9**, 656 (2020). 32855811 10.12688/f1000research.24680.1 PMC7425125

[57] Crozet, P. et al. Birth of a photosynthetic chassis: A MoClo toolkit enabling synthetic biology in the microalga Chlamydomonas reinhardtii. *ACS Synth. Biol.* **7** (9), 2074–2086 (2018). 30165733 10.1021/acssynbio.8b00251

[58] Baier, T., Wichmann, J., Kruse, O. & Lauersen, K. J. Intron-containing algal transgenes mediate efficient Recombinant gene expression in the green microalga Chlamydomonas reinhardtii. *Nucleic Acids Res.* **46** (13), 6909–6919 (2018). 30053227 10.1093/nar/gky532 PMC6061784

[59] Oldenhof, H., Zachleder, V. & Van Den Ende, H. Blue- and red-light regulation of the cell cycle in *Chlamydomonas reinhardtii* (Chlorophyta). *J. Phycol.* **41** (3), 313–320 (2006).

[60] Li, X. et al. An indexed, mapped mutant library enables reverse genetics studies of biological processes in Chlamydomonas reinhardtii. *Plant. Cell.* **28** (2), 367–387 (2016). 26764374 10.1105/tpc.15.00465 PMC4790863

[61] Hammond, T. G. et al. Cell spinpods are a simple inexpensive suspension culture device to deliver fluid shear stress to renal proximal tubular cells. *Sci. Rep.* **11** (1), 21296 (2021). 34716334 10.1038/s41598-021-00304-8 PMC8556299

[62] Hassler, D. M. et al. Mars’ surface radiation environment measured with the Mars science laboratory’s curiosity Rover. *Science* **343**, 1244797. 10.1126/science.1244797 (2014). 24324275 10.1126/science.1244797

