# Correction: Programmed cell death and redox metabolism protect *Chlamydomonas reinhardtii* populations from the galactic cosmic environment on the Artemis-1 mission

**DOI:** 10.1038/s41598-025-25139-5

**Published:** 2025-10-29

**Authors:** Timothy G. Hammond, Sajanlal R. Panikkanvalappil, Patricia L. Allen, Hamid Kian Gaikani, Corey Nislow, Guri Giaever, Ye Zhang, Howard G. Levine, Ramona Gaza, Dinah Dimapilis, Howard W. Wells, James M. Russick, Pierre M. Durand, Holly H. Birdsall

**Affiliations:** 1 Research Service Line, Durham VA Health Care System, Building 15, Room 210 508 Fulton Street, Durham, NC 27705 USA; 2 Nephrology Division, Department of Internal Medicine, Duke University School of Medicine, Durham, NC 27705 USA; 3 Department of Imaging, Dana-Farber Cancer Institute, Harvard Medical School, Boston, MA USA; 4 Department of Radiology, Brigham & Women’s Hospital, Harvard Medical School, Boston, MA USA; 5 Institute for Medical Research, Durham, NC 27705 USA; 6 Faculty of Pharmaceutical Sciences, The University of British Columbia, Vancouver, BC V6T 1Z3 Canada; 7 Kennedy Space Center, NASA-Kennedy Space Center, Utilization & Life Sciences Office, Merritt Island, FL 32899 USA; 8 Civil Group Integrated Missions Operation, Leidos, Houston, TX 77258 USA; 9 Space Radiation Analysis Group, NASA Johnson Space Center, Houston, TX 77058 USA; 10 Kennedy Space Center, The Bionetics Corporation, Mail code: Bio-2, Florida, FL 32899 USA; 11 Evolutionary Studies Institute, University of the Witwatersrand, Johannesburg, 2193 South Africa; 12 Departments of Otorhinolaryngology, Immunology, and Psychiatry, Baylor College of Medicine, Houston, TX 77030 USA; 13 Research Service Line, Durham VA Health Care System, Durham, NC 27705 USA

Correction to: *Scientific Reports* 10.1038/s41598-025-05419-w, published online 02 July 2025

The original version of this Article contained errors in Figure 3, where the colors representing “Flight” and “Ground” were swapped. “Flight” was erroneously identified as the green columns, and “Ground” was erroneously identified as the blue columns. The original Fig. 3 appears below.

**Fig. 3:**
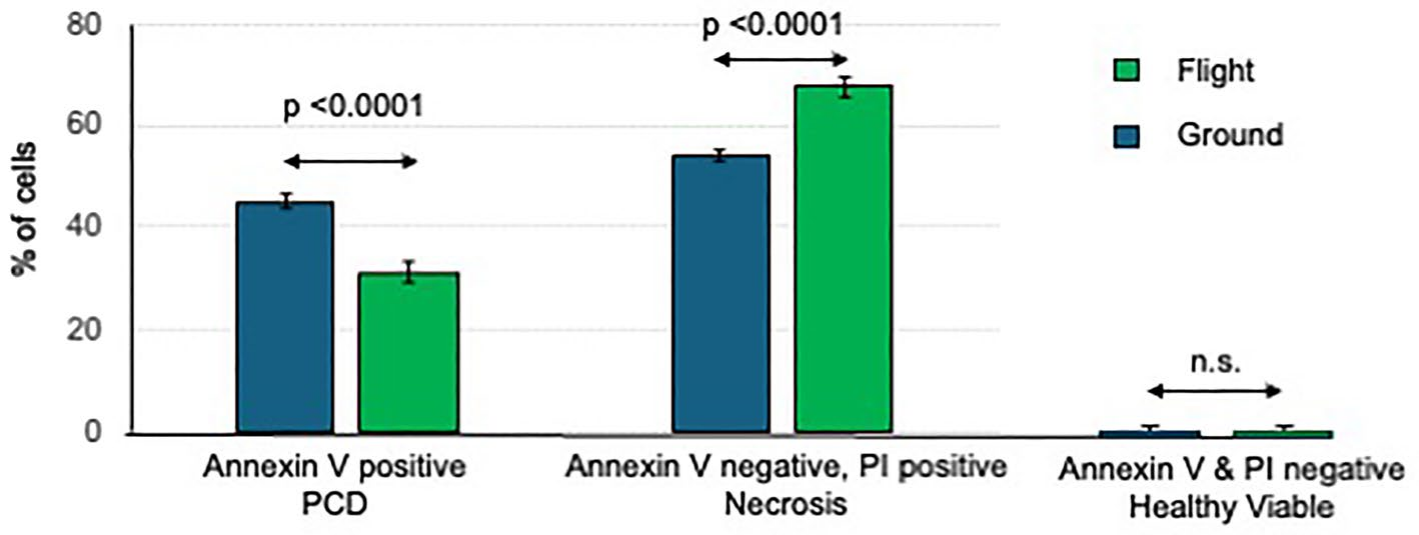
Viability and programmed cell death (PCD) of Chlamydomonas in flight vs. ground. Aliquots of flown and ground control Chlamydomonas were stained with Annexin V, to identify PCD, and propidium iodide, to identify dead cells, and analyzed by flow cytometry. Values shown are the % positively staining cells and are the mean ± SEM of six replicates. The p values for significance were evaluated by unpaired two-tailed t-test.

The original Article has been corrected.

